# Analysis of the factors that influence caregiver burden in adolescents with dual diagnosis

**DOI:** 10.1192/j.eurpsy.2023.1569

**Published:** 2023-07-19

**Authors:** S. Susana Perez, I. Martin-Herrero

**Affiliations:** Psychiatry, Los Arcos del Mar Menor Universitary Hospital, murcia, Spain

## Abstract

**Introduction:**

Serious mental illness in adolescence that not only has a significant impact on the patient but also on other contexts such as their family. Caregivers assume almost all of the care. This responsibility exposes caregivers to intense overload with negative consequences.

**Objectives:**

To evaluate and quantify the overload of the primary caregiver in children and adolescents with severe mental disorders.

**Methods:**

A prospective study was designed using structured interviews in caregivers of patients between 11 and 18 years of age with severe mental disorders and substance use who were evaluated in the psychiatric emergency service for 2 months. The Zarit Caregiver Burden Scale was used for quantitative assessment. Diagnosis, main caregiver, socio-health resources were recorded.

**Results:**

Of a total of 35 patients with serious mental illness between 11 and 16 years of age, the following was observed: intense caregiver burden in 42.2% of cases, moderate in 21%. Being the main caregiver the mother. 27.5% had adequate use of socio-health resources, while 42.7% had not requested them. The greatest need detected was economic and rehabilitation.

**Conclusions:**

Serious mental disorder present since adolescence leads families to significant destabilization of the family nucleus and comorbidity of psychiatric disorders in caregivers. Good orientation and evaluation of individual cases are necessary to guide families about the resources available in the social and health network, thus avoiding a high overload of caregivers and improving the quality of life of families.

**Disclosure of Interest:**

None Declared

